# Is polycystic ovary syndrome associated with uterine malformations? A systematic review using Bradford Hill’s causality framework

**DOI:** 10.1093/hropen/hoag005

**Published:** 2026-01-25

**Authors:** Stefano Palomba, Flavia Costanzi, Giuseppe Seminara, Donatella Caserta, Antonio Aversa

**Affiliations:** Department of Medical-Surgical Sciences and Translational Medicine, University “Sapienza” of Rome, Rome, Italy; Unit of Obstetrics and Gynecology, GOM of Reggio Calabria, Reggio Calabria, Italy; Department of Medical-Surgical Sciences and Translational Medicine, University “Sapienza” of Rome, Rome, Italy; Endocrinology Unit, “Renato Dulbecco” University Hospital, Catanzaro, Italy; Department of Experimental and Clinical Medicine, University “Magna Graecia” of Catanzaro, Catanzaro, Italy; Department of Medical-Surgical Sciences and Translational Medicine, University “Sapienza” of Rome, Rome, Italy; Unit of Gynecology, Sant’Andrea Hospital, Rome, Italy; Endocrinology Unit, “Renato Dulbecco” University Hospital, Catanzaro, Italy; Department of Experimental and Clinical Medicine, University “Magna Graecia” of Catanzaro, Catanzaro, Italy

**Keywords:** polycystic ovary syndrome, PCOS, infertility, pregnancy complications, uterus, malformations, Bradford Hill

## Abstract

**STUDY QUESTION:**

Is there a causal relationship between polycystic ovary syndrome (PCOS) and the occurrence of congenital uterine anomalies (CUAs)?

**SUMMARY ANSWER:**

Bradford Hill criteria did not support the causal relationship between PCOS and CUAs.

**WHAT IS KNOWN ALREADY:**

PCOS and CUAs are both linked to infertility and complications during pregnancy, but it is unclear whether PCOS increases the risk of developing CUAs.

**STUDY DESIGN, SIZE, DURATION:**

A systematic review with qualitative analysis using Bradford Hill criteria was performed.

**PARTICIPANTS/MATERIALS, SETTING, METHODS:**

Studies evaluating CUAs in women with PCOS or mechanisms linking PCOS-related factors to Müllerian development were selected. Comprehensive searches were conducted in MEDLINE, Web of Science, Cochrane Library, and Scopus for studies published in English from 1 January 2000 up to 30 August 2025 using terms related to PCOS, CUAs, and Müllerian anomalies. The nine Bradford Hill criteria were applied using modern epidemiological tools and concepts. For each Bradford Hill criterion, we prioritized studies according to the highest available level of evidence as defined by the Oxford Centre for Evidence-Based Medicine (CEBM). Study quality and risk of bias were systematically assessed using validated tools appropriate to each study design.

**MAIN RESULTS AND THE ROLE OF CHANCE:**

Twenty-one studies were included (5 systematic reviews with meta-analysis, 4 case–control, 11 cohort, 1 cross-sectional). Evidence supported the Bradford Hill criteria of biological gradient, coherence, plausibility, and analogy, whereas consistency, specificity, temporality, and experimental evidence were not met, and the strength of association only partially fulfilling. Overall, the findings provide weak qualitative support for a potential causal relationship between PCOS and CUAs.

**LIMITATIONS, REASONS FOR CAUTION:**

Findings are limited by heterogeneity in diagnostic criteria and imaging methods, and potential selection bias. In addition, the strength of association is characterized by large coefficient interval and modest significance.

**WIDER IMPLICATIONS OF THE FINDINGS:**

Routine uterine imaging for identifying/screening CUAs is not justified by the PCOS diagnosis alone until standardized prospective studies demonstrate a causal inference between diseases.

**STUDY FUNDING/COMPETING INTEREST(S):**

Departmental funds (Departments of Medical-Surgical Sciences and Translational Medicine, Sapienza University of Rome, and of Experimental and Clinical Medicine of ‘Magna Graecia’, University of Catanzaro; Italy) were used to support the authors throughout the study period and manuscript preparation. No specific external funding was received. The authors declare no competing interests.

**REGISTRATION NUMBER:**

PROSPERO CRD420251049920.

WHAT DOES THIS MEAN FOR PATIENTS? Polycystic ovary syndrome is a common condition in women that affects hormones, fertility, and pregnancy outcomes. Congenital uterine anomalies are differences in how the uterus develops before birth, and they can also make it harder to conceive and carry a pregnancy. We reviewed studies published over the past 25 years to explore whether these two conditions are linked. The application of the Bradford Hill criteria, a set of widely used scientific principles that help researchers decide whether one condition is likely to cause another, did not support the causal relationship between polycystic ovary syndrome and congenital uterine anomalies. In fact, the overall certainty of this association is very low because only few Bradford Hill criteria are satisfied. In addition, the studies available are small, differ widely from one another, vary in quality, and use different methods to make diagnosis of polycystic ovary syndrome and congenital uterine anomalies. Thus, the systematic and universal assessment of uterine morphology in women with polycystic ovary syndrome should be not recommended in the clinical practice.

## Introduction

Polycystic ovary syndrome (PCOS) is the most prevalent endocrine disorder among reproductive-age women with a prevalence of 5–10% worldwide (Bozdag *et al.*, 2016). This syndrome is associated with menstrual irregularities, hyperandrogenism, polycystic ovarian morphology (PCOM), and increased anti-Müllerian hormone (AMH) (Teede *et al.*, 2023). Women with PCOS have an impaired fertility (Palomba, 2021) and an increased risk of early and late pregnancy complications (Palomba *et al.*, 2015), even after adjustment for confounding factors such as age and BMI (Bahri Khomami *et al.*, 2024). That increased risk, in combination with its long-term cardiometabolic comorbidities, is a health condition that imposes a heavy economic burden on the health care system (Azziz *et al.*, 2005; Riestenberg *et al.*, 2022).

Congenital uterine anomalies (CUAs) (Grimbizis *et al.*, 2013), due to the deviation of formation, fusion, or resorption of the Müllerian duct in the uterus during fetal development (Moore *et al.*, 2008), have been also historically linked with infertility and adverse obstetric outcomes (Green and Harris, 1976; Acien, 1996; Raga *et al.*, 1997). These clinical associations have been recently confirmed (Hosseinirad *et al.*, 2021; Sallée *et al.*, 2022). However, these findings may be confounded by methodological constraints in estimating prevalence. In fact, the prevalence of CUAs is widely variable from 0.06% to 38% because of heterogeneous classification systems (Grimbizis *et al.*, 2013; Pfeifer *et al.*, 2021), different diagnostic methods employed (e.g. imaging, hysteroscopy, laparoscopy), and different cohorts and populations studied (Guimarães Filho *et al.*, 2006; Aslan *et al.*, 2022; Yavuz *et al.*, 2025).

A recent systematic review with meta-analysis (Zaborowska *et al.*, 2025) concluded that the available evidence about the uterine morphology and anomalies in women with PCOS is of very low-quality and a possible association between PCOS and some uterine anomalies may be only suggested. Moreover, women with PCOS may undergo more frequent and extensive gynecological evaluations—including ultrasound, hysterosalpingography, or hysteroscopy—due to increased infertility. This may lead to higher detection of CUAs compared with healthy women, in whom anomalies often remain undiagnosed. In consideration of the lack of well-established direct and close relationship between PCOS and CUAs, recent guidelines (Teede *et al.*, 2023; Palomba *et al.*, 2025) did not recommend the evaluation of the uterine morphology as a routine procedure for infertile patients with PCOS.

The use of Bradford Hill’s causality framework (Hill, 1965) is an epidemiological approach to identify the relationship between two variables, especially considering whether an observed association between a possible risk factor and a disease is truly causal. The Bradford Hill criteria remain a key tool in epidemiology for assessing whether an observed association between an exposure and an outcome is likely to be causal, more than just statistical correlation and indicates that there should be true etiological evidence. Their effectiveness has been demonstrated in major public health discoveries, most notably in establishing the causal link between cigarette smoking and lung cancer (Doll and Hill, 1950), and, over the years, it has been applied across a wide range of research fields (Howick *et al.*, 2019; Colebunders *et al.*, 2021; Nowinski *et al.*, 2022; Gomersall *et al.*, 2024). Recently, modern causal inference methods and new types of data have been integrated into the criteria (Schünemann *et al.*, 2011; Fedak *et al.*, 2015; Shimonovich *et al.*, 2021), to make them more flexible and applicable to current-day epidemiologic inquiry.

According to these observations, the aim of this study was to critically review and summarize the current knowledge using Bradford Hill’s causality framework to clarify if there is a causal link between PCOS and CUAs.

## Methods

This systematic review was conducted within the Preferred Reporting Items for Systematic Reviews and Meta-Analyses (PRISMA) guidelines (Page *et al.*, 2020). The protocol for the present review has been registered on the PROSPERO database (Protocol study registration code: PROSPERO CRD420251049920). Specific literature searches were performed for knowledge required to inform our assessments of the Bradford Hill criteria. The main comprehensive search of MEDLINE, Web of Science, Cochrane Library, and Scopus was conducted using the following terms: ‘androgens’ or ‘anti-Müllerian hormone’ or ‘AMH’ or ‘hyperandrogenism’ or ‘hyperinsulinemia’ or ‘insulin resistance’ or ‘obesity’ and ‘polycystic ovarian morphology’ or ‘polycystic ovary syndrome’ or ‘PCOS’ or ‘testosterone’ AND ‘malformations’ or ‘anomalies’ or ‘Müllerian ducts anomalies’. The full database-specific search strings are shown in Supplementary Table S1. The search was carried out from 1 January 2000 up to 30 August 2025.

Studies were eligible if they were systematic reviews with meta-analysis, observational (cohort, case–control, or cross-sectional) or experimental studies and if they compared CUAs in women with and without PCOS or investigated the relationship between PCOS, PCOS features/characteristics and Müllerian development. Non-English language studies, case series, and case reports were excluded. Retracted articles [checked through the Retraction Watch database of retracted articles (https://www.crossref.org/documentation/retrieve-metadata/retraction-watch/)] or articles subject to expressions of concern were also excluded. Two reviewers (F.C., G.S.) independently performed title/abstract screening, full-text review, and data extraction using a standardized extraction form that included population characteristics, index test, reference test, and outcomes. The full texts of the selected articles were revised, and incongruities were resolved by a third reviewer (S.P.) to confirm consistency in study selection.

For each Bradford Hill criterion, we prioritized studies according to the highest available level of evidence as defined by the Oxford Centre for Evidence-Based Medicine (CEBM) (http://www.cebm.ox.ac.uk/, accessed 21 September 2025). We first considered systematic reviews (with or without meta-analyses), followed by randomized controlled trials (RCTs), then prospective non-randomized or observational designs (cohort, case–control, or cross-sectional studies). Finally, we also examined translational, preclinical, and experimental studies when higher-level evidence was lacking. If multiple studies with the same level of evidence were identified, we selected the most recent publication exhibiting the lowest risk of bias. When overlapping studies of similar quality from the same year were encountered, both were included in the analysis. Study quality was assessed using the Methodological Quality of Systematic Reviews 2 (AMSTAR-2, Shea *et al.*, 2017, http://www.amstar.ca) for systematic reviews, Revised tool for Risk of Bias (rRoB 2, Sterne *et al.*, 2019, https://www.riskofbias.info/welcome/rob-2-0-tool) for RCTs, Risk Of Bias in Non-randomized Studies—of Intervention (ROBINS-I, Sterne *et al.*, 2016) for prospective non-randomized studies, and the Newcastle–Ottawa Scale (NOS, Wells *et al.*, 2014, http://www.ohri.ca/programs/clinical_epidemiology/oxford.asp) for observational/cohort studies.

The literature intercepted was analyzed to assess the causal association between PCOS and CUAs applying the nine Bradford Hill ‘viewpoints’ (Hill, 1965; Shimonovich *et al.*, 2024). For each ‘viewpoint’, the study/studies with highest quality was/were considered. Considering the specific nature of the review—aimed at identifying evidence to meet the Bradford Hill criteria—a quantitative synthesis was planned in case such data were missing in the literature (Schick-Makaroff *et al.*, 2016), to be conducted following the recommendations of the Cochrane Handbook (McKenzie and Brennan, 2024) and accompanied by an assessment of risk of bias due to missing results using the ROB-ME tool (Page *et al.*, 2023, 2024). The original ‘viewpoints’ (Hill, 1965) were integrated with modern epidemiological tools and concepts like Directed Acyclic Graphs (DAGs), Sufficient-Component Cause models (SCC), and the Grading of Recommendations Assessment, Development, and Evaluation (GRADE) methodology (Shimonovich *et al.*, 2021). Specifically, the following criteria were analyzed:

1. *Association strength*. To measure the strength of the association between PCOS and uterine malformations, we used the odds ratio (OR) and relative risk (RR), considering both the magnitude and precision of the observed association. According to established guidelines (Hill, 1965; Higgins *et al.*, 2024), a strong association—defined as a large effect size [e.g. RR or OR≥2] with adequate statistical precision—was considered less likely to be fully explained by confounding or bias. In contrast, modest or imprecise associations were not considered sufficient to satisfy this criterion. Each study was appraised for the risk of residual confounding, and potential sources of bias or unmeasured confounding were assessed through directed DAGs (Shimonovich *et al.*, 2021; Lesko and Fox, 2025). Certainty in the evidence (CoE) of the association was also evaluated based on the GRADE guideline, where it could be upgraded if a large effect size was present or downgraded in cases where a substantial risk of bias or confounding was suspected (Shimonovich *et al.*, 2021; Lesko and Fox, 2025).
2. *Consistency*. The consistency of the association was evaluated by examining common findings across multiple studies, diverse populations, clinical settings, and methodologies (Shimonovich *et al.*, 2021; Lesko and Fox, 2025). Consistency was considered as present when at least 50% of the included studies showed a relationship in the expected direction, as indicated in the forest plots. When forest plots were not available, we applied GRADE, with attention focused on residual inconsistency, as judged by the statistic heterogeneity (inconsistency measure, *I*^2^) (Higgins *et al.*, 2024). A rule of thumb was 50% or less, serving as a threshold before the overall criterion was met, specifically for analyses that pooled data across the greatest studies. Consistency minimizes the possibility of results being due to chance or context specific. In addition, the analysis aimed to assess whether consistent findings were also observed across countries, clinical settings, and population types, and to assess statistical heterogeneity across studies and the transportability of the findings, including structural differences between populations, such as the presence of additional risk factors (Shimonovich *et al.*, 2021; Lesko and Fox, 2025). For GRADE, any unexplained inconsistency (i.e. high heterogeneity, conflicting findings) resulted in downgrading the CoE.
3. *Specificity*. The specificity of the association between exposure (PCOS) and disease outcome (CUAs) was tested by assessing whether only one disease outcome occurred due to PCOS (Shimonovich *et al.*, 2021; Lesko and Fox, 2025). Here, specificity refers to observing the relationship for a single exposure, resulting in a single effect (Shimonovich *et al.*, 2021). Although high specificity is rare in reproductive epidemiology and complex disease status, it does not preclude the existence of a causal relationship (Lesko and Fox, 2025). To investigate this criterion, studies were searched for employing ‘negative control outcomes’ (malformations not associated with PCOS) or ‘negative control exposures’ (other conditions apart from PCOS, like infertility or miscarriage) to verify the possible existence of residual confounding. This criterion was considered fulfilled if the exposure–outcome relationship was highly specific (which is rare in many public health settings), or not if the exposure was linked to multiple outcomes, the outcome could be due to multiple exposures, or the specificity could not be demonstrated.
4. *Temporality*. Temporality was evaluated by asking whether PCOS (exposure) temporally precedes the development of CUAs (outcome), which is a fundamental and essential requirement for establishing any causal relationship in epidemiology (Shimonovich *et al.*, 2021; Lesko and Fox, 2025). The review was restricted to studies that intentionally confirmed this temporal direction, and preference was given to those employing prospective or matched case–control designs. This guaranteed that the diagnosis of PCOS predated the detection of uterine malformations and minimized the influence of reverse causality. Under this rule, temporality criterion was considered satisfied if study designs (e.g. prospective cohorts) clearly showed exposure to precede the outcome.
5. *Gradient or dose–response relationship*. A comparison of the risk of CUAs with the severity or persistent duration of PCOS assessed the dose–response relationship (Hill, 1965; Shimonovich *et al.*, 2021). The stability of these gradients was addressed, considering possible confounders, to minimize the potential for bias in any reported dose–response. The CoE was rated up according to the GRADE framework if a consistent dose–response association was shown (Shimonovich *et al.*, 2021; Lesko and Fox, 2025). According to principles of causal inference, a dose–response relationship with increasing levels of exposure will increase the probability that the association is real (Hill, 1965; Shimonovich *et al.*, 2021). If studies reported a gradient relation consistently (statistically or descriptively) the criterion was considered respected.
6. *Coherence*. Coherence refers to the degree of significant theoretical/scientific coherence between PCOS (exposure) and the development of CUAs (outcome) (Colvin *et al.*, 2018; Shimonovich *et al.*, 2021). The consistency of the evidence was determined by ensuring that the observed association was not inconsistent with existing relationship between maternal factors and CUAs (Hill, 1965; Colvin *et al.*, 2018; Shimonovich *et al.*, 2021). Literature was evaluated to identify if any inconsistent patterns in the prevalence or distribution of the CUAs are known (Colvin *et al.*, 2018). Coherence criterion was considered fulfilled if there were no conflicts with existing theory or empirical data and the overall integration of findings was strong. In contrast, in cases where contradictions, unresolved ambiguities, or alternative explanations were present the criterion was not fulfilled (Colvin *et al.*, 2018; Shimonovich *et al.*, 2021).
7. *Experimental evidence*. Evidence for effects was sought through identification of RCTs, quasi-RCTs or experimental studies on animal model evaluating an intervention (e.g. interruption, treatment) on prevalence or incidence of CUAs (Shimonovich *et al.*, 2021; Lesko and Fox, 2025). All studies were deemed to lead to higher CoE in terms of GRADE, since they have better control over confounding and bias. Evidence from experiments is especially prized in causal inference, as it demonstrates that changing the exposure results in the expected change in outcome (Schünemann *et al.*, 2011; Shimonovich *et al.*, 2021). In presence of direct/robust quasi-experimental evidence this criterion was considered respected.
8. *Plausibility*. This criterion was assessed by investigating whether there are established biological or physiological mechanisms that logically support a causal relationship between PCOS and the development of CUAs in offspring (Hill, 1965; Shimonovich *et al.*, 2021; Whaley *et al.*, 2022). This required a review of available preclinical information in relation to known pathophysiological bases for this association (Whaley *et al.*, 2022). For a causal relation to be plausible, the link must be consistent with what is known about biological or social mechanisms, and the evidence should be either experimental or observational. Valuation was based on pathways with a precise and well-established biological, physiological, or theoretical rationale (Shimonovich *et al.*, 2021).
9. *Analogy*. Analogy was considered by examining whether there are documented causal associations between other maternal endocrinopathies or female reproductive disorders and the occurrence of CUAs (Hill, 1965; Weed, 2018; Shimonovich *et al.*, 2021). This additional evidence can buttress causal inferences, although it does not largely drive them (Weed, 2018; Lesko and Fox, 2025). The principle of analogy for causation holds that, if one has observed biologically similar agents (e.g. related diseases, related drugs, related environmental exposures) with similar effects, then it is more plausible that the hypothesized objects or agents will have the same effect if the analogy is particularly close (Weed, 2018). When relevant analogies were found from closely related conditions or phenomena, the criterion was considered satisfied (Weed, 2018; Shimonovich *et al.*, 2021).

For each included association, the existence, or lack thereof, of each of the causality criteria was assessed through qualitative appraisal. Each criterion was discussed highlighting whether the available evidence could be interpreted as fulfilling, partially fulfilling, or not fulfilling that specific criterion. Papers included in the systematic review were grouped according to these individual criteria and data were assembled, which permitted the evidence to be considered in relation to the established causal model. Whenever possible, confounding factors (such as BMI, treatments, infertility status) were controlled and analyzed in the analyses.

## Results

**Figure 1.**
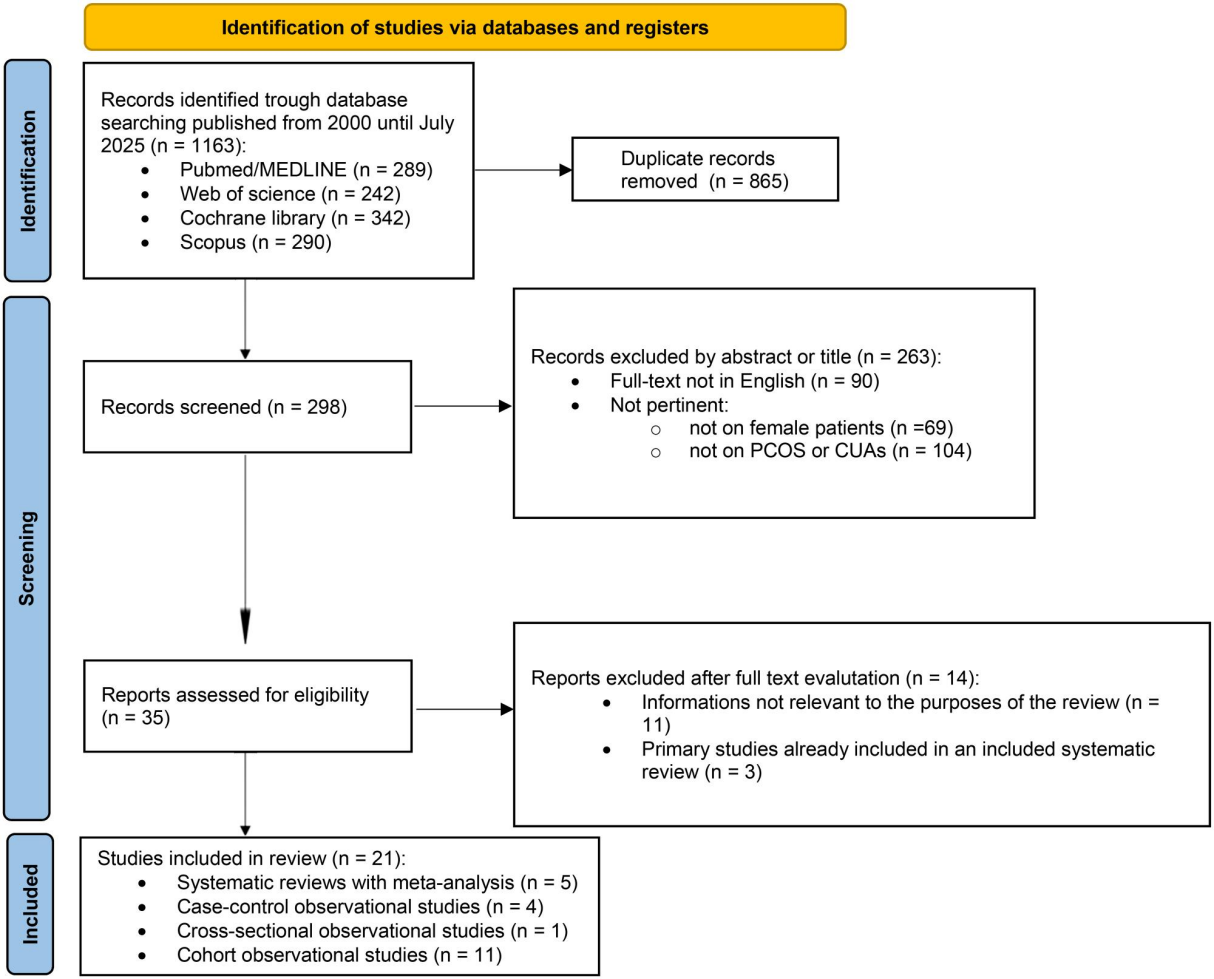
**PRISMA 2020 flow diagram.** CUAs, congenital uterine anomalies; PCOS, polycystic ovary syndrome.

A total of 1163 studies, published between 1 January 2000 and 30 August 2025, were identified through the literature search. Reading the title and abstract (and full text as necessary), 21 studies were included in the final analysis. Among these, 5 systematic reviews with meta-analysis, 4 case–control observational studies, 11 cohort observational studies, and 1 cross-sectional observational study were included, analyzed, and discussed in this review according to our interpretation of each of Bradford Hill’s criteria. The study selection process is summarized in the PRISMA flow diagram (Fig. 1). Supplementary Table S2 contains the studies excluded from the present review after full-text screening, along with the reasons for exclusion. Considering the little number of studies selected for each criterion—including a recent systematic review with meta-analysis (Zaborowska *et al.*, 2025)—no additional quantitative synthesis of the collected data was performed, and the findings were analyzed and reported in a qualitative fashion. Therefore, a formal assessment of risk of bias due to missing results was not performed. Nevertheless, during qualitative assessment, studies were examined for completeness of reporting and consistency between objectives, methods, and presented results.

The application of the Bradford Hill framework in the present review indicated that four of the nine criteria—biological gradient, coherence, plausibility, and analogy—were judged as fulfilled, while association strength, consistency, temporality, specificity, and experimental evidence were not supported by the data available in the literature. Taken together, these findings suggest weak qualitative evidence supporting a possible causal relationship between PCOS and CUAs, while acknowledging important limitations in study quality and design.

The analysis data for each specific Bradford Hill’s criterium has been below reported in a narrative fashion using the pre-defined methodology, followed by a discussion of the interpretation of studies’ results. Our interpretation of the Bradford Hill criteria for the purpose of this review is summarized in Table 1.

**Table 1.** Bradford-Hill criteria for assessing a causal association between PCOS and CUAs.

Criterion	Application in this review	Main references included	Results	Criterion assessment	**LoE** *	Assessment of quality
Association strength	Assigned in presence of a large effect size (RR or OR ≥ 2) with adequate statistical precision	Zaborowska *et al.* (2025)	OR 5.96, 95% CI 1.22–29.17	Partially assigned	3a	High^a^
Consistency	Assigned in presence of low-to-moderate heterogeneity (*I*²≤50%); not assigned in presence of a high heterogeneity (*I*²>50%)	Zaborowska *et al.* (2025)	*I*² 97.56%	Not assigned	3a	High^a^
Specificity	Assigned if the exposure-outcome relationship is highly specific (i.e. PCOS associated with only one CUA and not with multiple anomalies; or demonstrated via negative control experiments); not assigned if PCOS is linked to multiple outcomes or if outcomes may have multiple exposures	Zaborowska *et al.* (2025)	PCOS was associated with different CUAs. No study employed systematic negative control outcomes or exposures. Outcome-specificity was not demonstrated, and controls often included women with other risk factors (e.g. infertility, hormonal disorders).	Not assigned	3a	High^a^
		Chan *et al.* (2011)	Higher prevalence of several CUAs was also observed in infertile, miscarrying, and unselected women, indicating that these anomalies are shared across different at-risk groups and are not specific to PCOS		2a	Moderate^a^
Temporality	N/A			Not assigned	N/A	N/A
Gradient	Assigned if the increasing level of exposure to androgen and/or AMH levels corresponds to a higher risk of CUAs or specific types of CUAs in patients with PCOS	Aslan *et al.* (2022)	In the PCOS subgroup, free testosterone levels were positively correlated with the presence of CUA (*P *= 0.03). Using a threshold of 2.025 ng/ml, free testosterone levels predicted CUAs, with an AUC of 0.765 (95% CI 0.569–0.960, *P *= 0.03)	Assigned	2b	High^b^
		Yang *et al.* (2023)	In patients with PCOS, the prevalence of unicornuate uterus was higher in the high-AMH group (>8.45 ng/ml) compared to the low-AMH group (1.0% vs 0.1%, *P *= 0.04). No significant difference was observed in the overall incidence of uterine malformations (4.3% vs 5.7%, *P *= 0.22)		2b	Moderate^b^
Coherence	Assigned if the association is consistent with existing biological and clinical knowledge; we considered the criterion supported in presence of convergence of reproductive outcomes observed across diverse studies, particularly in the setting of pregnancy	Bahri Khomami *et al.* (2024)/Venetis *et al.* (2014)	Women with PCOS and CUAs have both an increased risk of miscarriage (OR 1.49, 95% CI 1.20–1.85, *I*^2^ 82.8% and RR 1.68, 95% CI 1.31–2.15, *I*^2^ 89%, respectively)	Assigned	3a	High^a^/Low^a^
		Bahri Khomami *et al.* (2024)/Panagiotopoulos *et al*. (2022)	Women with PCOS and CUAs have both an increased risk of PTB (OR 1.53, 95% CI 1.33–1.75, *I*^2^ 67.6% and OR 3.89, 95% CI 3.11–4.88, *I*^2^ 75%, respectively).		3a	High^a^/Moderate^a^
Experimental evidence	Assigned in presence of experimental or intervention-based evidence to support the association	N/A		Not assigned	N/A	N/A
Plausibility	Assigned in presence of a biological, physiological, or theoretical rationale explaining the association	N/A		Assigned	N/A	N/A
Analogy	Assigned when relevant analogies were found from conditions closely related to PCOS	Tokhunts *et al.* (2022)	I-shaped uterine anomaly detected exclusively in patients with hyperandrogenic endocrinopathies (24.3% in patients with PCOS and 39.5% in patients with CAH); not detected in patients with other endocrine causes of infertility (0%)	Assigned	3b	Moderate^b^

AMH, anti-Mullerian hormone; CAH, congenital adrenal hyperplasia; CUAs, congenital uterine anomalies; *I*^2^, heterogeneity; LoE, level of evidence; N/A, not applicable; PCOS, polycystic ovary syndrome; PTB, preterm birth; OR, odds ratio; RR: relative risk.

a Methodological Quality assessed with Systematic Reviews 2 (AMSTAR-2, http://www.amstar.ca, accessed 21 September 2025).

b Methodological Quality assessed with Newcastle-Ottawa Scale (NOS, Wells *et al.*, 2014, http://www.ohri.ca/programs/clinical_epidemiology/oxford.asp, accessed 21 September 2025).

* Level of evidence defined by the Oxford Centre for Evidence-Based Medicine (CEBM) (http://www.cebm.ox.ac.uk/, accessed 21 September 2025).

### Association strength

In this review, the criterion of strength of association refers to the magnitude of the observed relationship between PCOS and CUAs, with stronger associations indicating a higher likelihood of a potential causal link. The search for the highest-quality evidence on association between PCOS and CUAs identified data synthesis from three case–control studies and two cross-sectional studies involving 7285 patients (1308 PCOS vs 5977 controls) (Zaborowska *et al.*, 2025).

That meta-analysis found a greater probability of CUAs in patients with PCOS (25.0%, 95% CI 6.9–43.2) compared to infertile non-PCOS women (5.3%, 95% CI 0.5–14.8) (Zaborowska *et al.*, 2025). Using the Mantel–Haenszel random effects model, the observed result resulted statistically significant with an OR of 5.96 (Zaborowska *et al.*, 2025). However, the association was not considered precise in consideration of the large CI ranging from 1.22 to 29.17 and of modest significance (*P *= 0.028). The level of evidence by CEBM is 3b considering that it is a systematic review/meta-analysis of case–control and cross-sectional studies (Zaborowska *et al.*, 2025) and that it is downgraded for the substantial risk of bias or confounding, as below discussed (Shimonovich *et al.*, 2021; Lesko and Fox, 2025).

Even if the methodological quality assessment (AMSTAR-2, Shea *et al.*, 2017) indicated moderate quality (Zaborowska *et al.*, 2025), there is not sufficient evidence to completely support the Bradford Hill criterion of the strength of association now.

### Consistency

The Bradford Hill criterion of consistency evaluates whether the observed association is similar across different studies and populations. In our literature review, the highest quality meta-analysis highlighted very high heterogeneity across studies (*I*^2^ = 97.56%) (Zaborowska *et al.*, 2025), indicating substantial variability in effect estimates. This level of heterogeneity prevents reliable conclusions regarding consistency, as the results are not uniform across the included studies. The level of evidence by CEBM is 3b (see above) (Zaborowska *et al.*, 2025) and the methodological quality assessment (AMSTAR-2, Shea *et al.*, 2017) indicated moderate quality (Zaborowska *et al.*, 2025). Therefore, the criterion of consistency cannot be considered fulfilled based on the currently available evidence.

### Specificity

The specificity criterion of Bradford Hill tests whether the relationship between PCOS and CUAs is limited to a specific type of malformation, indicating a direct and specific cause. A recent systematic review investigated whether PCOS may influence some specific CUAs and uterine morphology by meta-analysis (Zaborowska *et al.*, 2025). The results revealed a statistically significant relationship between PCOS and specific CUAs. In fact, a higher risk of uterine septum (OR 4.21, 95% CI 1.44–12.31, *P *= 0.009) and of didelphys uteri (OR 10.72, 95% CI 1.57–73.20, *P *= 0.016) was detected in women with PCOS when compared with controls (Zaborowska *et al.*, 2025). The prevalence of uterine septum was 18.7% (95% CI 3.5–33.9) and 5.9% (95% CI 0.3–17.9) for PCOS and control group, respectively (7 studies including 1804 vs 7394, respectively, for PCOS and control subjects) (Zaborowska *et al.*, 2025). Similarly, the prevalence of didelphys uteri was 0.3% (95% CI 0.05–0.7) and 0.0001% (95% CI 0–0.09) for PCOS and control group, respectively (2 studies including 1119 vs 5814, respectively, for PCOS and control subjects) (Zaborowska *et al.*, 2025). On the other hand, no association was identified between PCOS and arcuate uterus, T-shaped uterus, bicornuate, or unicornuate uterus (Zaborowska *et al.*, 2025).

Critically, to explore the criterion of specificity and the possibility that other exposures might also be implicated (‘negative control exposures’) in the etiology of CUAs, other evidence-based data were searched. A systematic review (Chan *et al.*, 2011) was intercepted and here analyzed/discussed. This work systematically assessed the prevalence of CUAs in women with infertility (independent of PCOS), with miscarriage, and in unselected population, providing an indirect negative control by demonstrating that a higher prevalence of some CUAs (particularly canalization and unification defects) was shared by different at-risk groups and was not explicitly linked to PCOS (Chan *et al.*, 2011). The level of evidence was classified as 2a according to the CEBM, and the methodological quality (AMSTAR-2, Shea *et al.*, 2017) deemed moderate (Chan *et al.*, 2011).

The level of evidence by CEBM is 3b (see above) (Zaborowska *et al.*, 2025), and the methodological quality assessment (AMSTAR-2, Shea *et al.*, 2017) indicated moderate quality (Zaborowska *et al.*, 2025).

Overall, these data (Chan *et al.*, 2011; Zaborowska *et al.*, 2025) suggest that an increased risk of the two CUAs is possible in PCOS, particularly the septate and didelphys uterus, but that PCOS is not restricted to one specific malformation type, with no evidence of a monopoly of any anomaly in PCOS. In addition, the existence of associations with other forms of malformations and the multitude of malformation nomenclature systems makes the criterion of specificity less relevant. According to these points, the criterion of specificity was considered not met.

### Temporality

According to causality certainties, one of the fundamental criteria that should be met is temporality. Regarding the relationship between PCOS and CUAs, it is relevant that CUAs are congenital conditions, resulting from abnormal development of the Müllerian ducts during fetal life, and are present at birth. In contrast, PCOS is usually diagnosed after puberty when symptoms and signs associated with hyperandrogenism, ovulatory dysfunction, and PCOM have developed. This temporal disjunction means that, strictly speaking, the temporal criterion for causality cannot be satisfied by current evidence, as the potential ‘exposure’ (*in utero* factors predisposing to PCOS) and the ‘outcome’ (CUAs) do not follow a classic exposure–outcome timeline.

The crucial causal question, therefore, is whether the intrauterine hormonal environment that may predispose offspring to PCOS also plays a role in the etiology of abnormal Müllerian development resulting in CUAs. However, all studies available to date are retrospective or cross-sectional, and none have prospectively evaluated prenatal exposures and the subsequent development of both PCOS and CUAs in offspring (Chan *et al.*, 2011; Ege *et al.*, 2019; Fujii and Oguchi, 2023; Zaborowska *et al.*, 2025). It is essential to recognize this limitation when interpreting all reported associations between PCOS and CUAs in the literature, as doing so ensures methodological rigor in the assessment of causality. Consequently, it is not possible to establish a temporal sequence or definitive causal direction between PCOS and CUAs based on the available evidence. Thus, the criterion of temporality in relation to the association of PCOS and CUAs was not met.

### Gradient or dose–response relationship

The gradient or dose–response criterion implies that, if a causal association exists, an increasing level of exposure should correspond to a higher risk of the outcome. PCOS is a heterogeneous condition, and no universally accepted measure of ‘severity’ exists. Consequently, it is not possible to define a dose–response relationship based on overall PCOS severity. To address this limitation, in the present review, we considered several studies that used biological markers as indicators for the intensity of the syndrome. Circulating androgen levels and AMH concentrations have been considered indicative of the degree of endocrine dysfunction (Laven, 2025).

One retrospective cohort study reported that serum free testosterone levels were significantly higher in patients with CUAs, both in the total population and specifically in the subgroup of PCOS patients, classified according to ESHRE/European Society for Gynaecological Endoscopy (ESGE) criteria (Aslan *et al.*, 2022). In the PCOS subgroup, a positive correlation was observed between free testosterone levels and the presence of CUAs (*P *= 0.03) (Aslan *et al.*, 2022). Using a threshold of 2.025 ng/ml, free testosterone showed a sensitivity of 87.5% and a specificity of 59% for predicting CUAs, with an AUC of 0.765 (95% CI 0.569–0.960, *P *= 0.03) (Aslan *et al.*, 2022). These findings highlight that higher androgen levels are associated with an increased likelihood of CUAs. However, the overall methodological quality assessment was low (assessed with the NOS, Wells *et al.*, 2014) (Aslan *et al.*, 2022). According to CEBM, the study qualifies as level 2b evidence (retrospective cohort study).

In a retrospective cohort study of 1391 women with PCOS, participants were stratified into a low-AMH group (n = 700) and a high-AMH group (n = 691) using a cutoff of 8.45 ng/ml (Yang *et al.*, 2023). The study found that the prevalence of unicornuate uterus was higher in the high-AMH group compared to the low-AMH group (1.0% vs 0.1%, *P *= 0.04), although no significant difference was observed in the overall incidence of CUAs between the two groups (4.3% vs 5.7%, *P *= 0.22) (Yang *et al.*, 2023). These findings suggest that elevated AMH levels may be associated with a higher risk of specific Müllerian anomalies in women with PCOS, particularly unicornuate uterus. The methodological quality by the NOS (Wells *et al.*, 2014) was moderate (Yang *et al.*, 2023). According to CEBM, this study is also rated as level 2b evidence (retrospective cohort study) (Yang *et al.*, 2023).

Taken together, these findings provide support for the biological gradient criterion, suggesting that higher levels of androgens and/or AMH—used as indicators of PCOS severity—are associated with an increased risk of specific uterine malformations.

### Coherence

The coherence criterion of Bradford Hill refers to whether evidence from different disciplines produces complementary findings that fit together logically. In this context, the association between PCOS and CUAs is supported by the convergence of reproductive outcomes observed across diverse studies, particularly in the setting of pregnancy.

The rate of miscarriage is significantly higher in both conditions. A meta-analysis of 44 studies demonstrated a significantly increased risk of miscarriage in women with PCOS (OR 1.49; 95% CI 1.20–1.85; *I*^2^ = 82.8%) (Bahri Khomami *et al.*, 2024). The AMSTAR-2 (Shea *et al.*, 2017) quality assessment indicated high quality (Bahri Khomami *et al.*, 2024). Similarly, meta-analytic data derived from 14 studies found a higher risk of miscarriage, both in first than in second trimester, in women affected by CUAs than in controls (RR 1.68; 95% CI 1.31–2.15; *I*^2^ = 89%) (Venetis *et al.*, 2014). Unfortunately, the quality assessment (AMSTAR-2, Shea *et al.*, 2017) indicated low quality (Venetis *et al.*, 2014).

Similarly, the risk of preterm birth (PTB) is also increased in both conditions. Indeed, a meta-analysis of 54 studies reported comparatively a higher PTB in the offspring of women with PCOS when compared with non-PCOS controls (OR 1.53; 95% CI 1.33–1.75; *I*^2^ 67.6%) (Bahri Khomami *et al.*, 2024). Another meta-analysis of 15 studies (Panagiotopoulos *et al*., 2022) demonstrated that the risk of PTB is nearly four times higher in women with CUAs than in control women without CUAs (OR 3.89; 95% CI 3.11–4.88; *I*^2^ = 75%). The AMSTAR-2 (Shea *et al.*, 2017) quality assessment indicated moderate quality (Panagiotopoulos *et al*., 2022). All referenced meta-analyses are classified as CEBM level 3a evidence, since they are systematic reviews or meta-analyses of observational studies-not randomized trials.

The convergence of these meta-analytic findings highlights a pattern of similar adverse reproductive events, i.e. consisting of increased risks of miscarriage and PTB, in both conditions. This alignment of evidence from different studies and clinical contexts emphasizes how the observed reproductive complications fit together and are not incoherent from a Bradford Hill perspective. Thus, the coherence criterium about the causal association between PCOS and CUAs was met.

### Experimental evidence

The criterion of experimental evidence does not seem to be applicable to the association between PCOS and CUAs. After extensive review of the literature, we did not identify any RCT, quasi-randomized trial, or interventional study in which exposure to PCOS was considered as an ‘intervention’ to evaluate subsequent risk of CUAs. Likewise, we found no experimental animal studies employing established models of PCOS, such as prenatal hyper-androgenization, that specifically investigated the occurrence of CUAs as an outcome. Similarly, there is no experimental study evaluating the (beneficial) effect of anti-androgens on the risk of CUAs. This absence of experimental data prevents any direct inference of causality based on this criterion. At present, the available evidence is therefore limited to observational epidemiological studies and the association between PCOS and CUAs cannot be supported by interventional or experimental evidence. Thus, the experimental evidence criterium was considered not met.

### Plausibility

The plausibility criterion refers to the extent to which an observed association can be explained by existing biological or mechanistic knowledge. From a biological standpoint, plausibility is a key criterion for interpreting the association between PCOS and CUAs. It implies the existence of a coherent pathophysiological mechanism that can explain epidemiological observations, grounded in established knowledge of endocrinology and embryology. PCOS hormonal imbalances are largely programmed during fetal life by genetic and environmental factors, particularly *in utero* androgen exposure (Barsky *et al.*, 2021). Translational studies demonstrate that prenatal androgen exposure, as observed in daughters of women with PCOS, can alter ovarian gene expression and mitochondrial function at birth, affecting folliculogenesis, oocyte maturation, and ovarian metabolism (Barsky *et al.*, 2021).

Experimental data support that excess androgens and/or abnormal AMH levels during critical windows of Müllerian duct formation may alter also uterine morphogenesis (Cheng *et al.*, 2011; Moramezi *et al.*, 2013; Mullen and Behringer, 2014; Aslan *et al.*, 2020). The androgen-dependent theory of uterine anomalies has been widely explored (Panidis *et al.*, 2014; Aslan *et al.*, 2022; Tokhunts *et al.*, 2022; Fujii and Oguchi, 2023). Prolonged androgen exposure has been suggested as a potential disruptor of uterine development (Tokhunts *et al.*, 2022). Both androgens and AMH can modulate the activity of developmental genes, including the expression of the homeobox (*HOX*) and wingless-related integration site (*WNT*) genes, which regulate Müllerian duct differentiation and patterning (Cheng *et al.*, 2011; Mullen and Behringer, 2014; Aslan *et al.*, 2022). Furthermore, metabolic alterations common in PCOS, such as insulin resistance, type 2 diabetes mellitus, and impaired uterine perfusion, could interfere with intrauterine development and condition functional uterine changes in adulthood (Schaefer-Graf *et al.*, 2000; Chekir *et al.*, 2005). Similarly, a study noted that reduced uterine volume was associated with more severe hyperandrogenemia in classic PCOS phenotypes, likely due to decreased uterine blood perfusion (Panidis *et al.*, 2014). However, genetic susceptibility and inheritance patterns of PCOS may additionally explain the overlap between endocrine dysfunction and CUAs (Kahsar-Miller *et al.*, 2001; Vink *et al.*, 2006; Coviello *et al.*, 2011).

Taken together, the current literature shows that the association between PCOS and CUAs is plausible, suggesting common underlying mechanisms potentially related to prenatal androgen exposure. Thus, the plausibility criterion is substantially satisfied, as theoretical pathogenetic rationale may link these conditions.

### Analogy

The criterion of analogy was explored by evaluating whether CUAs are also associated with reproductive endocrinopathies other than PCOS, particularly those that share overlapping features of hyperandrogenism. The analysis of literature revealed two main conditions affecting potentially the risk of CUAs and having similar characteristics of PCOS; androgen-secreting tumors in pregnancy and congenital adrenal hyperplasia (CAH), an established hyperandrogenic state of adrenal origin. No data was intercepted regarding the relationship between androgen-secreting tumors in pregnancy and the CUAs risk, whereas interesting studies were identified about the influence of CAH on uterine embryogenesis.

A case–control study conducted on 486 patients investigated the prevalence of I-shaped uterus, a subtype of T-shaped uterus, in women presenting with infertility or recurrent miscarriage (Tokhunts *et al.*, 2022). Data stratified by endocrine profile: 74 women with PCOS, 43 women with CAH and 45 women with other endocrinopathies (e.g. thyroid function abnormalities, hyperprolactinemia, hyper- and hypogonadotropic amenorrhea, and premature ovarian failure) (Tokhunts *et al.*, 2022). Notably, this anomaly was detected exclusively in patients with hyperandrogenic disorders. In fact, I-shaped uterus was diagnosed in 24.3% and 39.5% of patients with PCOS and with CAH, respectively, whereas no cases were observed in infertile patients without PCOS/CAH (Tokhunts *et al.*, 2022). The case–control study (Tokhunts *et al.*, 2022) was assessed as moderate quality (NOS; Wells *et al.*, 2014) and classified as level 3b evidence (CEBM criteria).

The identification of CUAs across both PCOS and CAH is particularly relevant, as these conditions share common endocrine pathways, including elevated androgen levels and ovulatory dysfunction. By analogy, if CAH is causally associated with I-shaped uterine anomalies, then it is plausible that PCOS may exert similar developmental effects. These converging findings across related endocrine disorders provide supportive evidence that strengthens the association between PCOS and CUAs and supports the analogy criterion from a Bradford Hill perspective. Thus, the analogy criterium was considered met.

## Discussion

The present systematic review is the first study aimed to evaluate whether a causal association between PCOS and CUAs using a well-structured epidemiologic approach. Overall, in line with the predefined methodological strategy, our qualitative analysis revealed that the available evidence supports only four of the nine Bradford Hill criteria, i.e. biological gradient, coherence, plausibility, and analogy. Consistency, specificity, temporality, and experimental evidence were not fulfilled, whereas the strength of association, the main indicator of causal association in the modern science, was only partially fulfilled. In particular, the strength of association, one of the main epidemiological criteria, was not completely fulfilled notwithstanding a high OR for CUAs in women with PCOS. In fact, the CIs were very large and the inconsistency was high, reducing the weight of the criterium. This balance suggests that even if several elements may point toward a potential etiological relationship, the Bradford Hill criteria did not support the causal relationship between PCOS and CUAs with certainty.

In interpreting these findings, it is important to consider the conceptual nature of the Bradford Hill framework. The evaluation of its criteria is not intended as a numerical or summative exercise but rather as a structured, interpretive process aimed at guiding causal reasoning. Although quantitative weighting may appear to enhance clarity and has been used in similar studies applied to other contexts (Gomersall *et al.*, 2024), it risks oversimplifying complex relationships and obscuring the relative importance of individual viewpoints. Some criteria, such as temporality, represent essential prerequisites for causality, whereas others, like analogy, serve as supportive contextual evidence. A qualitative, narrative assessment therefore offers a more faithful application of the framework and better captures the nuanced character of the current evidence base.

The strongest signal emerged from the strength of association criterion, where pooled data demonstrated significantly higher odds of CUAs in women with PCOS compared to controls, particularly for septate and didelphys uterus (Zaborowska *et al.*, 2025). However, the evidence was accompanied by wide CIs and modest significance with low certainty ratings. The findings of the most recent meta-analysis also underscored the poor quality of existing studies (Zaborowska *et al.*, 2025). Importantly, the observed high heterogeneity (*I*^2^>90%) diminishes the reproducibility of these findings across populations. Thus, while the observed effect sizes are notable, they should be interpreted with caution. However, the meta-analysis intercepted and analyzed (Zaborowska *et al.*, 2025) did not include the most recent cohort study on the topic (Yavuz *et al.*, 2025). This last study (Yavuz *et al.*, 2025) included a total of 297 patients, comprising 99 women with PCOS and 198 healthy controls. The results confirmed the association between PCOS and CUAs according to the American Society for Reproductive Medicine (ASRM) (Pfeifer *et al.*, 2021) and ESHRE/ESGE criteria (Grimbizis *et al.*, 2013, 2016) (17.2% vs 3%, *P *< 0.0001; and 10.1% vs 2%, *P *= 0.003, respectively) (Yavuz *et al.*, 2025). Therefore, it is not possible to predict whether the inclusion of this study would have modified the observed OR and *I*^2^.

The selection of control populations is a significant methodological concern. As shown in the results section, most studies recruited infertile women as controls, a subgroup with an intrinsically higher prevalence of CUAs than the general female population. This methodological design may lead to a bias toward the null, potentially masking the true magnitude of the association between PCOS and CUAs. On the other hand, women with PCOS might receive gynecological examinations more often than healthy peers, which would contribute to CUAs’ overrepresentation in this group. The concurrent existence of these counteracting biases may in part account for the high inter-study heterogeneity and inconsistent effect estimates.

According to Bradford Hill’s original conceptualization, strength and consistency represent distinct and independent viewpoints: the former refers to the size of an observed effect, while the latter assesses the reproducibility of that effect across different contexts and studies. In this sense, inconsistency does not automatically nullify the presence of an apparently strong association but rather qualifies and moderates its contribution within the overall causal appraisal. Consequently, in our qualitative synthesis, the strength of association was cautiously considered supportive, yet heavily qualified by the profound uncertainty introduced by interstudy variability.

Coherence represents a nuanced concept within the Bradford Hill framework. In our approach, we focused on the reproductive outcomes associated with both PCOS and CUAs, evaluating coherence as the absence of evidence contradicting the observed association. Rather than requiring a strict mechanistic overlap, coherence was assessed in terms of whether the association aligns with current biological understanding of how these conditions impact reproductive outcomes. This perspective acknowledges that, although the underlying causal pathways may differ, the observed link between PCOS and CUAs is not inconsistent with known effects on fertility, implantation, and pregnancy outcomes.

Overall, when compared to filtered previous evidence (Zaborowska *et al.*, 2025), our qualitative analysis according to Bradford Hill criteria did not confirm the possible causal association between PCOS and CUAs, reinforcing the low methodological certainty of the available literature. Both our analysis and prior syntheses converge on the conclusion that current data are fragmented, often based on retrospective or cross-sectional designs, and substantially limited by inconsistent diagnostic criteria for both PCOS and CUAs. This methodological heterogeneity complicates attempts at generalizability.

This current study has several strengths. To our knowledge, it is the first attempt to systematically appraise the association between PCOS and CUAs through the lens of causal inference, explicitly applying all nine Bradford Hill criteria. The use of predefined methodological hierarchies validated quality assessment tools, and the application of the GRADE framework enhances transparency and reproducibility. The structured approach also allowed us to identify specific gaps in knowledge that impede a stronger causal claim.

Nevertheless, the quality of the available evidence was low. Because most included studies were observational and 8 out of 11 were judged to be of poor quality, the overall certainty was downgraded for high risk of bias, confounding, and heterogeneity (Zaborowska *et al.*, 2025). Several methodological weaknesses contributed to this. Assessment techniques varied widely, ranging from two-dimensional ultrasonography, saline infusion sonography, hysterosalpingography, hysteroscopy, and laparoscopy, to MRI, which was used in only four studies (Zaborowska *et al.*, 2025). Such variability, coupled with the use of different classification systems for uterine anomalies [American Fertility Society 1988 (American Fertility Society, 1988), ASRM 2021 (Pfeifer *et al.*, 2021), ESHRE/ESGE (Grimbizis *et al.*, 2013, 2016), Congenital Uterine Malformations by Experts 2019 (Ludwin *et al.*, 2018, 2020), or non-standard definitions], introduces substantial misclassification and compromises comparability. This explains the very large CIs and considerable heterogeneity observed in the pooled estimates. Moreover, inadequate assessment methods and lack of harmonization across classification systems remain pervasive limitations in studies of uterine anomalies.

Selection bias represents an additional concern. Patients with PCOS undergo more frequent and extensive imaging investigations due to increased infertility prevalence, leading to a higher detection of CUAs compared with non-PCOS controls. Similarly, in most studies, the control groups consisted primarily of infertile patients rather than a truly general non-PCOS population. Since CUAs are more prevalent in infertile women than in the general population (8.0% vs 5.5%) (Chan *et al.*, 2011), using such a control group introduces systematic bias toward the null. This means the observed association may significantly overestimate or underestimate, respectively, the true effect of PCOS on the risk of CUAs.

However, a few studies excluded from previous meta-analytic synthesis (Zaborowska *et al.*, 2025) have suggested a possible link between isolated PCOM and Müllerian anomalies (Macdougall *et al.*, 1992; Ugur *et al.*, 1995; Appelman *et al.*, 2003), which may warrant further investigation. The inability to adequately account for infertility status, PCOS phenotypes, and hormonal treatments in both cases and controls further complicates interpretation, and their clinical implications should be also considered with caution.

From a clinical point of view, current evidence does not justify routine and universal assessment of uterine morphology in all women with PCOS, confirming recent guideline recommendations (Teede *et al.*, 2023; Palomba *et al.*, 2025). However, in selected clinical contexts—such as women with PCOS presenting with recurrent miscarriage or repeated ART failure—targeted imaging may be warranted to exclude structural anomalies that could adversely affect outcomes.

From a research perspective, several priorities emerge. Future studies should adopt prospective designs to establish temporality and minimize bias. Both cases and controls should be rigorously defined, with a formal diagnosis of PCOS required for inclusion in cases, and PCOS features, and hormonal treatments carefully excluded in controls. Moreover, the choice of imaging methods is crucial. High-resolution techniques that allow for visualization of both the uterine cavity and external contour, such as MRI or three-dimensional ultrasonography, should be favored to increase diagnostic accuracy while reducing invasiveness and cost. Harmonization of classification systems is equally essential to improve comparability across studies. Finally, large, multicenter cohorts with careful adjustment for confounders such as BMI, metabolic status, and infertility history will be necessary to clarify the magnitude and specificity of the association.

In conclusion, this systematic review applying the Bradford Hill framework provides a structured qualitative assessment of the potential causal link between PCOS and CUAs. Bradford Hill criteria did not support the causal relationship between PCOS and CUA in consideration that only four out of nine criteria were fully satisfied. However, the overall certainty of evidence was very low, hampered by methodological heterogeneity, risk of bias, and total lack of temporality or experimental data. Current findings should not alter routine clinical practice, but they highlight the urgent need for carefully designed prospective studies using standardized diagnostic approaches. Clarifying this association has the potential to improve reproductive risk stratification and clinical management in women with PCOS.

## Supplementary Material

hoag005_Supplementary_Data

## Data Availability

All data used in this systematic review are derived from previously published studies that are publicly available through the databases and sources described in the Materials and Methods section. No new data were generated or analysed.
