# Supplementary material for: Is polycystic ovary syndrome associated with uterine malformations? A systematic review using Bradford Hill’s causality framework
Source: Hum Reprod Open. 2026 Jan 25;2026(1):hoag005. doi: 10.1093/hropen/hoag005 (PMC12902784; doi:10.1093/hropen/hoag005)
Supplement: hoag005_Supplementary_Data [file hoag005_supplementary_data.zip › Supplementary Table S1.docx]

**Supplementary Table S1**. Search strategy for each database.

| **Database** | **Search string** |
| --- | --- |
| Pubmed/MEDLINE | ("Androgens"[Mesh] OR androgens[tiab] OR "Anti-Mullerian Hormone"[Mesh] OR "anti-Mullerian hormone"[tiab] OR AMH[tiab] OR "Hyperandrogenism"[Mesh] OR hyperandrogenism[tiab] OR "Hyperinsulinism"[Mesh] OR hyperinsulinemia[tiab] OR "Insulin Resistance"[Mesh] OR "insulin resistance"[tiab] OR "Obesity"[Mesh] OR obesity[tiab] OR "Testosterone"[Mesh] OR testosterone[tiab]) AND ("Polycystic Ovary Syndrome"[Mesh] OR "polycystic ovary syndrome"[tiab] OR PCOS[tiab] OR "Polycystic Ovarian Morphology"[tiab]) AND ("Mullerian Ducts/abnormalities"[Mesh] OR "Congenital Abnormalities"[Mesh] OR malformations[tiab] OR anomalies[tiab] OR "Müllerian duct anomalies"[tiab]) |
| Web of Science | TS=((Androgen* OR "anti-mullerian hormone" OR AMH OR Hyperandrogen* OR  hyperinsulin* OR "insulin resistance" OR obes* OR testosterone*) AND ("polycystic ovary syndrome" OR PCOS OR "polycystic ovarian morpholog*") AND ("mullerian duct abnormalit*" OR "congenital abnormalit*" OR malformation* OR anomal* OR "mullerian duct anomal*")) |
| Cochrane library | Androgens OR androgen OR androgens OR Anti-Mullerian Hormone OR anti mullerian hormone OR AMH OR Hyperandrogenism OR hyperandrogenism OR Hyperinsulinism OR hyperinsulinemia OR Insulin Resistance OR insulin resistance OR Obesity OR obesity OR Testosterone OR testosterone AND Polycystic Ovary Syndrome OR polycystic ovary syndrome OR PCOS OR polycystic ovarian morphology AND Congenital Abnormalities OR Mullerian Ducts OR malformation OR malformations OR anomaly OR anomalies OR uterine anomaly OR uterine anomalies |
| Scopus | TITLE-ABS-KEY (androgen* OR "anti mullerian hormone" OR AMH OR hyperandrogenism OR hyperinsulinism OR hyperinsulinemia OR "insulin resistance" OR obesity OR testosterone) AND TITLE-ABS-KEY ("polycystic ovary syndrome" OR PCOS OR "polycystic ovarian morphology") AND TITLE-ABS-KEY ("mullerian duct anomaly" OR "mullerian duct anomalies" OR "mullerian duct anomaly" OR "mullerian duct anomalies" OR "congenital abnormality" OR "congenital abnormalities" OR malformation OR malformations OR anomaly OR anomalies OR "uterine anomaly" OR "uterine anomalies") |
