# Supplementary material for: Is polycystic ovary syndrome associated with uterine malformations? A systematic review using Bradford Hill’s causality framework
Source: Hum Reprod Open. 2026 Jan 25;2026(1):hoag005. doi: 10.1093/hropen/hoag005 (PMC12902784; doi:10.1093/hropen/hoag005)
Supplement: hoag005_Supplementary_Data [file hoag005_supplementary_data.zip › Supplementary Table S2 Revised.docx]

**Supplementary Table S2**. References excluded from the systematic review after full-text screening, with exclusion reasons.

|  | **Reference** | **Exclusion reason** |
| --- | --- | --- |
| 1 | Albdairi and Al-Shalah, 2021 | Primary study already included in an included systematic review |
| 2 | Saleh and Shawky Moiety, 2014 | Primary study already included in an included systematic review |
| 3 | Leonhardt et al., 2012 | Primary study already included in an included systematic review |
| 4 | Wang et al., 2013 | Assessment of CUAs not included |
| 5 | Franik and Skałba, 2011 | Assessment of CUAs not included |
| 6 | Pall et al., 2010 | Assessment of CUAs not included |
| 7 | Azziz et al., 2004 | Assessment of CUAs not included |
| 8 | Abraham Gnanadass et al., 2021 | Assessment of CUAs not included |
| 9 | Sanchez-Garrido and Tena-Sempere, 2020 | Assessment of CUAs not included |
| 10 | Zore et al., 2019 | Assessment of CUAs not included |
| 11 | Pinola et al., 2017 | Assessment of CUAs not included |
| 12 | Yang et al., 2016 | Assessment of CUAs not included |
| 13 | Ciresi et al., 2016 | Assessment of CUAs not included |
| 14 | Jamil et al., 2016 | Assessment of CUAs not included |

**References**

Abraham Gnanadass S, Divakar Prabhu Y, Valsala Gopalakrishnan A. Association of metabolic and inflammatory markers with polycystic ovarian syndrome (PCOS):an update. *Arch Gynecol Obstet* 2021;*303*: 631-643.

Albdairi AAH, Al-Shalah MAN. Cross-sectional study of the association between polycystic ovary syndrome and uterine septum anomalies. *Int J Pharm Res* 2021;*13*: 114–118.

Azziz R, Sanchez LA, Knochenhauer ES, Moran C, Lazenby J, Stephens KC, Taylor K, Boots LR. Androgen excess in women: experience with over 1000 consecutive patients. *J Clin Endocrinol Metab* 2004;*89*: 453-62.

Ciresi A, Amato MC, Bianco J, Giordano C. Prevalence and clinical features of polycystic ovarian syndrome in adolescents with previous childhood growth hormone deficiency. *J Pediatr Endocrinol Metab* 2016;*29*: 571-8.

Franik G, Skałba P. Clinical observations and hormone screenings of patients with non-standard hypertrophy of the adrenal cortex. *Endokrynol Pol* 2011;*62*: 230-7.

Jamil AS, Alalaf SK, Al-Tawil NG, Al-Shawaf T. Comparison of clinical and hormonal characteristics among four phenotypes of polycystic ovary syndrome based on the Rotterdam criteria. *Arch Gynecol Obstet* 2016;*293*: 447-56.

Leonhardt H, Gull B, Kishimoto K, Kataoka M, Nilsson L, Janson PO, Stener-Victorin E, Hellstrom M. Uterine morphology and peristalsis in women with polycystic ovary syndrome. *Acta Radiol* 2012;*53*: 1195–1201.

Pall M, Azziz R, Beires J, Pignatelli D. The phenotype of hirsute women: a comparison of polycystic ovary syndrome and 21-hydroxylase-deficient non classic adrenal hyperplasia. *Fertil Steril* 2010;*94*: 684-9.

Pinola P, Puukka K, Piltonen TT, Puurunen J, Vanky E, Sundström-Poromaa I, Stener-Victorin E, Lindén Hirschberg A, Ravn P, Skovsager Andersen M et al. Normo- and hyperandrogenic women with polycystic ovary syndrome exhibit an adverse metabolic profile through life. *Fertil Steril* 2017;*107*: 788-795.e2.

Saleh HA, Shawky Moiety FM. Polycystic ovarian syndrome and congenital uterine anomalies: the hidden common player. *Arch Gynecol Obstet* 2014;*290*: 355-360.

Sanchez-Garrido MA, Tena-Sempere M. Metabolic dysfunction in polycystic ovary syndrome: Pathogenic role of androgen excess and potential therapeutic strategies. *Mol Metab* 2020;*35*: 100937.

Wang ET, Kao CN, Shinkai K, Pasch L, Cedars MI, Huddleston HG. Phenotypic comparison of Caucasian and Asian women with polycystic ovary syndrome: a cross-sectional study. *Fertil Steril* 2013;*100*: 214-8.

Yang R, Yang S, Li R, Liu P, Qiao J, Zhang Y. Effects of hyperandrogenism on metabolic abnormalities in patients with polycystic ovary syndrome: a meta-analysis. *Reprod Biol Endocrinol* 2016;*14*: 67.

Zore T, Lizneva D, Brakta S, Walker W, Suturina L, Azziz R. Minimaldifference in phenotype between adolescents and young adults with polycysticovary syndrome. *Fertil* *Steril* 2019;*111*: 389-396.
